# Comparing Pool‐seq, Rapture, and GBS genotyping for inferring weak population structure: The American lobster (*Homarus americanus*) as a case study

**DOI:** 10.1002/ece3.5240

**Published:** 2019-05-26

**Authors:** Yann Dorant, Laura Benestan, Quentin Rougemont, Eric Normandeau, Brian Boyle, Rémy Rochette, Louis Bernatchez

**Affiliations:** ^1^ Institut de Biologie Intégrative et des Systèmes (IBIS) Université Laval Québec Canada; ^2^ Pêches et Océans Canada Institut Maurice‐Lamontagne Mont‐Joli Canada; ^3^ Plateforme d'analyses génomiques, Institut de Biologie Intégrative et des Systèmes (IBIS) Université Laval Québec Canada; ^4^ Department of Biology University of New Brunswick Saint John Canada

**Keywords:** GBS, Homarus, marine genomics, Pool‐seq, population genetics, Rapture

## Abstract

Unraveling genetic population structure is challenging in species potentially characterized by large population size and high dispersal rates, often resulting in weak genetic differentiation. Genotyping a large number of samples can improve the detection of subtle genetic structure, but this may substantially increase sequencing cost and downstream bioinformatics computational time. To overcome this challenge, alternative, cost‐effective sequencing approaches, namely Pool‐seq and Rapture, have been developed. We empirically measured the power of resolution and congruence of these two methods in documenting weak population structure in nonmodel species with high gene flow comparatively to a conventional genotyping‐by‐sequencing (GBS) approach. For this, we used the American lobster (*Homarus americanus*) as a case study. First, we found that GBS, Rapture, and Pool‐seq approaches gave similar allele frequency estimates (i.e., correlation coefficient over 0.90) and all three revealed the same weak pattern of population structure. Yet, Pool‐seq data showed *F*_ST_ estimates three to five times higher than GBS and Rapture, while the latter two methods returned similar *F*_ST_ estimates, indicating that individual‐based approaches provided more congruent results than Pool‐seq. We conclude that despite higher costs, GBS and Rapture are more convenient approaches to use in the case of species exhibiting very weak differentiation. While both GBS and Rapture approaches provided similar results with regard to estimates of population genetic parameters, GBS remains more cost‐effective in project involving a relatively small numbers of genotyped individuals (e.g., <1,000). Overall, this study illustrates the complexity of estimating genetic differentiation and other summary statistics in complex biological systems characterized by large population size and migration rates.

## 1 INTRODUCTION

Over the last decade, next‐generation sequencing (NGS) technologies have enhanced the development of population genomic studies in nonmodel organisms (Armengaud et al., 2014; Ekblom & Galindo, 2011; Ellegren, 2014; Narum, Buerkle, & Davey, 2013). The advent of NGS technology led to the occurrence of new molecular methods for population genomics analysis such as restriction site‐associated DNA Sequencing (RAD‐seq) or genotyping‐by‐sequencing (GBS); these methods have substantially increased the amount of genomic information available with thousands of single nucleotide polymorphisms (SNPs) being genotyped (Andrews, Good, & Miller, 2016). Using large genomic SNP datasets, both methods have shown significant improvement in our capacity to resolve fine‐scale population structure compared to microsatellites markers (Ferchaud, Laporte, Perrier, & Bernatchez, 2018; Malenfant, Coltman, & Davis, 2015; Vendrami et al., 2017). Moreover, these methods have enhanced the accuracy of demographic inference (Le Moan, Gagnaire, & Bonhomme, 2016; Rougemont et al., 2017; Shafer, Gattepaille, Stewart, & Wolf, 2015).

Uncovering population genomic structure of species characterized by large effective population size and/or high migration rate may be challenging since this often translates into a lack or very weak genetic differentiation and spatial genomic structure (Gagnaire et al., 2015; Holliday et al., 2017; Neale & Kremer, 2011; Waples, 1998). Recent studies suggested that increasing the number of samples and markers genotyped can improve the detection of subtle genetic structure in nonmodel species such as the polar bear (Viengkone et al., 2016), the candlefish (Candy et al., 2015), the American lobster (Benestan et al., 2015), the silvery lightfish (Rodriguez‐Ezpeleta, Álvarez, & Irigoien, 2017), the Tasmanian devil (Hendricks et al., 2017), or the sea cucumber (Xuereb et al., 2018). Yet, the genotyping of a large number of samples and markers may vary widely depending on the selected NGS protocol. Therefore, choosing the most appropriate NGS genotyping approach sometimes remains challenging. Roughly speaking, the lower the extent of genetic differentiation is, the higher the number of required samples and markers is to obtain narrow confidence intervals (CI) around estimates of genetic differentiation (Patterson, Price, & Reich, 2006). This may substantially increase the analytical cost and computational time (Shendure & Aiden, 2012). To overcome this challenge, alternative protocols to the classic way of sequencing individuals separately (i.e., each individual is sequenced with a unique barcode) have been developed either by pooling DNA samples such as Pool sequencing (Futschik & Schlötterer, 2010; Lynch, Bost, & Wilson, 2014; Schlötterer, Tobler, Kofler, & Nolte, 2014) or by reducing genomic complexity using sequence capture methods (Ali et al., 2016; Boucher, Casazza, Szövényi, & Conti, 2016; Hoffberg et al., 2016; Jones & Good., 2016).

Despite these promising alternatives, each approach has its own strengths and weaknesses, related to the distribution of polymorphic loci, the cost of library preparation and sequencing, and the accuracy of variant calling and genotyping. All of these factors may ultimately affect demographic inferences (Cutler & Jensen, 2010; Harvey, Smith, & Glenn, 2016). For instance, Pool sequencing (hereafter Pool‐seq) does not provide individual genotypes, whereas this information is essential for some applications such as assignment tests and linkage disequilibrium estimation (Cutler & Jensen, 2010). On the other hand, and pending on specific research objectives, quantifying genetic parameters where individual information is required may not always be necessary. In such cases, Pool‐seq has already proven to be an effective and accurate approach to investigate genome‐wide variations of terrestrial and marine high gene flow species such as oaks (*Quercus* spp., Leroy et al., 2018), poplar (*Populus alba*, Stölting et al., 2015; *Populus alba*, *Populus tremula*, Christe et al., 2016), Chinese chestnut (*Castanea mollissima*, LaBonte, Zhao, & Woeste, 2018), sticklebacks (*Gasterosteus aculeatus*, Guo, DeFaveri, & Sotelo, 2015), Atlantic herring (*Clupea harengus*, Guo, Li, & Merilä, 2016; Lamichhaney et al., 2012; Martinez Barrio et al., 2016), Atlantic cod (*Gadus morhua*, Karlsen et al., 2013), and the copepod (*Tigriopus californicus*, Lima & Willett, 2018). This latter approach was also successful to detect selection in the model species *Drosophila* spp. characterized by very large effective population size (e.g., Barghi et al., 2018; Bastide et al., 2013; Kapun et al., 2014). Furthermore, Pool‐seq offers the possibility to genotype a large number of individuals at a much lower cost than individual sequencing.

In contrast to Pool‐seq, sequence capture approaches enable the sequencing of a large number of samples while preserving genotypic information at the individual level (Andrews et al., 2016). Originally, this latter method targets known genomic regions such as exons, which limits the number and diversity of DNA sequences being studied (Harvey et al., 2016). For the past few years, sequence capture approaches have undergone a new upswing, in particular with the concept of targeted sequence enrichment that couples the power of sequence capture with NGS technology (Grover, Salmon, & Wendel, 2012). More recently, methods combining sequence capture enrichment and reduced representation libraries have been proposed (Ali et al., 2016; Boucher et al., 2016; Hoffberg et al., 2016; Suchan et al., 2016). In this study, due to its ease of use with GBS libraries, we focused our work on the so‐called Rapture protocol, which represents a highly flexible genotyping method protocol allowing thousands of individuals to be sequenced simultaneously with a high sequencing depth (Ali et al., 2016). However, this method requires known genomic sequences of interest (e.g., reference genome or targeted sequence information) in order to design capture probes (Ali et al., 2016; Jones & Good, 2016). The design of capture probes is a critical step since it may influence the quality of all the genomic data collected. Furthermore, this step can be potentially costly in terms of probes development and synthesis. Although targeted loci should be selected according to the experimental needs of a given project, this selection step may be hampered by the occurrence of paralogous or highly polymorphic sequences (Ali et al., 2016).

In sum, each of these NGS protocols (GBS, Pool‐seq, and Rapture) offers different benefits and limitations and all may be relevant to the field of population genomics in species exhibiting low genetic differentiation. To date, there is no study that have already explored and compared their relative efficiency in resolving weak population structure in nonmodel species. In this context, the goal of this study was to assess the consistency of two cost‐effective sequencing approaches, Pool‐seq and Rapture, in documenting genetic population structure as well as estimating allele frequency and derived statistics in a high gene flow and nonmodel species, the American lobster (*Homarus americanus*), comparatively to a conventional GBS approach.

## 2 MATERIALS AND METHODS

### 2.1 Sampling material and DNA extraction

**Figure 1:**
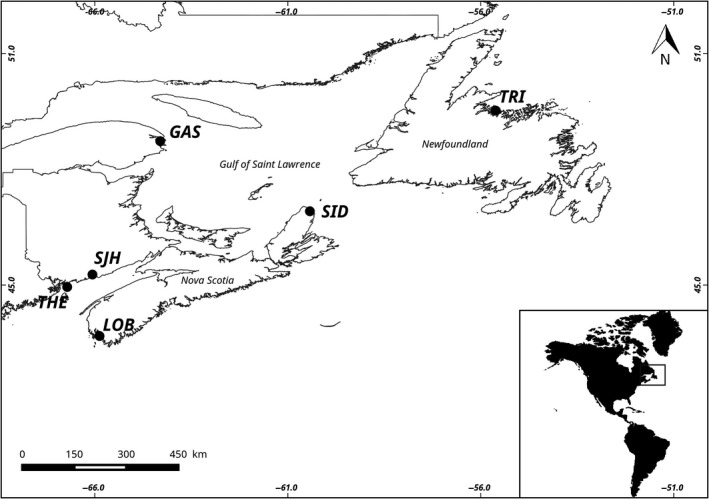
Map of lobster sampling locations. GAS, Gaspé; LOB, Lobster bay; SID, Sidney Bight; SJH, Saint‐John Harbour; THE, The Wolves/Deer island; TRI, Triton

A total of 288 egg‐bearing lobster females were collected between May and August 2012, from six locations (*N* = 48 individuals per location) across the Northeast Atlantic (Figure 1; see Benestan et al., 2015 for details). Half of the second walking leg of each individual was collected and preserved in 95% EtOH until DNA extraction. A previous study on the American lobster using RAD‐seq revealed the existence of both genetic structure and significant isolation by distance (IBD) (Benestan et al., 2015). The authors identified two main distinct units as (a) southern region (i.e., from USA Maine to midsouth of Nova Scotia shelf) and as (b) northern region (i.e., from midnorth of Nova Scotia shelf to the north of Newfoundland, including all the Gulf of St. Lawrence samples). Given this, we selected six sampling sites spread over each of these two pre‐identified regions (i.e., three northern sites, Gaspé (GAS), Sidney Bight (SID), and Triton (TRI), and three southern sites, Lobster bay (LOB), Saint‐John Harbour (SJH), and The Wolves/Deer island (THE); Figure 1). Genomic DNA was extracted using salt extraction (Aljanabi & Martinez, 1997) with an additional RNAse treatment following the manufacturer protocol. Genomic DNA quality was checked on 1% agarose gel, and specimens with too many smears (i.e., indicating degradation of DNA) were excluded from the entire dataset. Genomic DNA was then quantified using a NanoDrop instrument, roughly diluted, and final DNA concentrations were normalized to 20 ng/µl based on fluorescence reads values (AccuClear™ Ultra High Sensitivity dsDNA Quantitation Solution).

### 2.2 Library preparation

Individual GBS library was prepared following Mascher, Wu, and Amand (2013) and detailed in Moore et al. (2017). Briefly, genomic DNA was double‐digested using the *PstI* and *MspI* restriction enzymes followed by ligation to a unique barcoded adapter for each individual. For GBS‐based libraries, each individual was labeled with a unique barcode and 96 individuals were pooled for size selection, PCR, and sequencing (see sequencing details below).

For Pool‐seq library preparation, we used 2 µl DNA of equimolar concentrations for each individual of a given location. The 48 individuals of a given sampling site were pooled and barcoded using the same nucleotide sequence to identify sample origin. Here, the sample size of pooled DNA samples is a critical parameter, which will ultimately influence the accuracy of allele frequency estimates (Anderson, Skaug, & Barshis, 2014; Fracassetti, Griffin, & Willi, 2015; Futschik & Schlötterer, 2010; Gautier et al., 2013; Lynch et al., 2014; Rode et al., 2017; Schlötterer et al., 2014). Therefore, maximizing the number of samples contributing to the pool (Schlötterer et al., 2014) can minimize variance at the individual level potentially caused by technical errors (e.g., pipetting, DNA concentration estimations) or quality of samples (e.g., tissues or DNA quality). Moreover, several authors advocates that replication of pools may help to reduce the error rate in SNP calling (Gautier et al., 2013; Schlötterer et al., 2014). Here, our pool size (i.e., 48 samples) was selected according to Schlötterer et al. (2014) who suggested a sampling size ranging from 40 to 100 individuals analyzed. Then, we prepared three to four 48 samples pool replicates for each sampling site (depending on the availability of DNA) in order to control for experimental reproducibility and potential biases derived from Pool‐seq. Additionally, Pool‐seq technical replicates were used to compute average allele frequencies and pairwise *F*_ST_ values per sampling site in order to compare with statistical estimators derived from individual‐based datasets. Pool‐seq libraries were digested using the same restriction enzymes and protocol described above for GBS‐based libraries. We sequenced 16 pool libraries in a first sequencing Ion Proton chip containing four sampling sites (i.e., 4xGAS, 4xLOB, 4xSID, 4xTRI) and then six pool libraries in a second chip composed of the two remaining sampling sites (i.e., 3xSJH and 3xTHE).

Rapture sequencing was performed following Ali et al. (2016). First, custom probes were designed from a de novo reference catalog of 9,818 loci genotyped during the previous GBS library sequencing run (see details below). The probe library was purchased from Arbor Biosciences™, and we followed the Mybait protocol supplied with the capture kit. In order to explore the potential offered by this method that aims at reducing the sequencing costs relative to a conventional RAD‐seq approach, we increased the multiplexing load from 96 individual barcodes for our GBS library setup to 384 individual barcodes on our Rapture experiment. For these Rapture libraries, a total of 288 individuals from the six sampling sites used in this study were coupled with 93 others samples (required for another project) and three free‐DNA water blanks (used for sequencing/bioinformatic plate control) to sequence one Rapture library with similar sequencing efforts compared to the GBS and Pool‐seq libraries.

### 2.3 Library sequencing

All libraries were sequenced on the Ion Torrent p1v3 chip at the plateforme d'analyses génomiques of the Institute of Integrative and Systems Biology (IBIS, Université Laval, Québec, Canada) with a median target of 80 million single‐end reads (50–220 pb) per chip. Two rounds of sequencing (i.e., two separate chips) were conducted for all libraries. GBS libraries were normalized after the first round in order to reduce the unbalanced sequence representation of individuals by adjusting DNA volumes for each sample. Pool‐seq libraries normalization was not possible because individual information was unavailable, and therefore, balanced contribution of each individual in each pool is assumed. Rapture protocol was also conducted without normalization as the adjustment of DNA volumes on a highly randomized and multiplexed Rapture setup (i.e., several hundreds to one thousand barcodes) is very time‐consuming and could substantially increase the risk of inadvertent pipetting errors.

### 2.4 Data processing

#### 2.4.1 Construction of a de novo reference catalog of individual genotyping‐by‐sequencing (GBS) libraries

Genotyping‐by‐sequencing sequence data from four locations (Gaspé, Lobster Bay, Sidney Bight, Triton) were analyzed using the pipeline available at (https://github.com/enormandeau/stacks_workflow) according to Benestan et al. (2016). First, reads were trimmed to 80bp and shorter reads were discarded using cutadapt (Martin, 2011). Samples were then demultiplexed using *process_radtags* in STACKS V.1.38 (Catchen, Hohenlohe, & Bassham, 2013). A maximum of three nucleotide mismatches (*M* = 3), a minimum stack depth of three (*m* = 3), and a maximum distance for secondary reads *N* = 5 were allowed in *ustacks*. Then, reads were aligned de novo to create a catalog of putative loci (*cstacks* module in stacks,with default parameters) and the *populations* command was run requiring a locus to be present in at least one sampling location and in 50% of all individuals. Finally, this dataset was postfiltered using a custom python script (available at https://github.com/enormandeau/stacks_workflow/00-scripts/05_filter_vcf.py) where we kept SNPs for which a genotype was called in at least 70% of individuals in each sampling site with a Ho < 0.6 and a *F*_IS_ between [−0.7: 0.7]. A minor allele frequency (MAF) threshold of at least 1% globally or 5% in each sampling locality was also applied and no more than eight SNPs per locus were allowed. Based on this final individual GBS dataset, we then generated a targeted sequences catalog for Rapture. Ultimately, we removed highly similar sequences through a “self‐blast” test and RepeatMasker 4.0 (Smit, AFA, Hubley, R & Green, P. *RepeatMasker Open‐4.0*.2013‐2015 http://www.repeatmasker.org), respectively. The final panel was composed of 16,780 SNPs spread over 9,818 loci.

#### 2.4.2 Individual variant calling (GBS and Rapture)

Both GBS and Rapture raw data were processed using the same workflow as indicated previously for trimming and demultiplexing. Individual reads were aligned to the reference catalog with BWA‐mem (Li & Durbin, 2009) using default settings values except for minimum seed length (−k 19), maximum seed occurrence (−c 500), gap open penalty (−O 0), gap extension penalty (−E 2), and the output alignment score option disabled (−T 0). The resulting SAM files were then filtered to remove unmapped reads and perform secondary alignment as well as supplementary alignment using SAMtools view (Li et al., 2009). Then, reads with mapping quality less than 20 and reads containing soft clipping (i.e., the exclusion of terminal bases with mismatches) were removed. SNPs were identified using *pstacks* module specifying a 5× minimum depth coverage for each stack. This threshold was selected based on the reads distribution of each sample and in order to limit false‐positive SNPs resulting from sequencing errors. Then, a catalog was built using *cstacks* with three mismatches allowed between samples tags. We ran *populations* module requiring again a locus to be present in at least one population and at a frequency >50% in that population, with a minimal depth of five to be processed. The final dataset was obtained by keeping SNPs genotyped in at least 70% of the individuals in each sampling site, showing an observed heterozygosity < 0.6 within samples and a global MAF > 0.01. Since our de novo reference catalog was already filtered for *F*_IS_ to remove ambiguous SNPs, we did not apply this filter again on the mapped datasets. Genotype missing data threshold was set to 16% in order to retain more than 90% of individuals in both GBS and Rapture datasets.

#### 2.4.3 Pool‐seq variant calling

Pool‐seq sequences were trimmed, demultiplexed, and aligned across the reference catalog as previously described. Sequences Alignment/Map files in binary format (BAM) were then filtered as above, removing all reads with soft clipping (Kofler, Orozco‐terWengel, et al., 2011a). Then, BAM files were combined to generate a synchronized multiple pileup file using SAMtools mpileup tool (Li et al., 2009) and the Popoolation2 java script *mpileup2sync.jar* (Kofler, Pandey, & Schlötterer, 2011b) with default parameters. SNP calling was performed using the *popsync2pooldata* function in the R package *poolftsat* 0.0.1 (Hivert, Leblois, Petit, Gautier, & Vitalis, 2018). We considered only biallelic SNPs called with a minimal read count ≥ 4. We also required a minimal coverage of 30 and maximal coverage of 300 in order to remove poor‐quality SNPs and potential sequencing artifacts (i.e., PCR duplicates), respectively. Finally, we fixed a MAF threshold of at least 0.01 in each pool.

### 2.5 Assessing consistency between methods

#### 2.5.1 Testing for correlations among allele frequencies

We first identified shared SNPs between GBS, Rapture, and Pool‐seq datasets based on locus name information, locus read position, and SNP alleles. Allele frequencies for individual‐based data were computed for each sampling site using vcftools v0.1.12b (Danecek et al., 2011), for both GBS and Rapture VCF datasets. Pool‐seq allele frequency estimates were performed by dividing the read count of each SNP allele on the total locus coverage. Correlations of minor allele frequencies were calculated between each of the three methods for each shared SNP. We also computed the average MAF across all Pool‐seq replicates for each SNP to further reduce the potential bias of MAF estimations and to accurately examine the relationship between Pool‐seq, GBS, and Rapture methods using the Pearson correlation coefficient available in R under the *cor.test* function.

#### 2.5.2 Computing genetic differentiation for the three methods

First, the extent of genetic differentiation was computed and compared relatively to each method and to the entire dataset (i.e., using SNPs shared between all methods and the overall set of discovered SNPs in each method). We computed pairwise *F*_ST_ values between each sampling site using the *θ* estimator of Weir and Cockerham (1984). In order to minimize the effects of linkage disequilibrium, downstream analyses were performed using only one SNP per locus, by keeping only SNP showing the higher MAF at each locus. This last filtering step is expected to reduce the number of low‐frequency SNPs. Indeed, these rare variants are typically hard to distinguish from sequencing errors and mapping artifacts in low coverage NGS data without reference genome. Moreover, Guo et al. (2013) also demonstrated by simulations that Pool‐seq is not ideal for estimating allele frequencies of rare SNPs.

For GBS and Rapture, *F*_ST_ were computed using the *stamppFst* function from the R package StAMPP 1.5.1 (Pembleton, Cogan, & Forster, 2013) with 95% CI estimated on 1,000 bootstraps. Pool‐seq *F*_ST_ values were computed with the *computeFST* function available in the R package poolfstat 0.0.1, using the method of moments developed by Hivert et al. (2018). Briefly, this latter method is based on an analysis of variance derived from the Weir and Cockerham (1984) estimator and corrected for Pool‐seq datasets. CI for Pool‐seq *F*_ST_ was obtained using a custom bash script over 1,000 bootstraps iterations. Pool technical replicates were used to compute the average of each pairwise *F*_ST_ and CI values. Additionally, for all pairwise site comparisons, we performed standard Mantel tests to assess correlation between genetic distances (measured as *F*_ST_/(1 − *F*_ST_); Rousset, 1997) and geographic distances. Seafloor distances were measured between each sites using the R package *marmap* 0.9.6 (Pante & Simon‐Bouhet, 2013). This R toolbox enabled us to estimate marine distances along coast lines. Mantel test was performed with *Ade4* 1.7.10 (Dray & Dufour, 2007) using 1,000 permutations assuming a two‐dimensional habitat in which geographic distance was log‐transformed.

An additional analysis of allele frequency differentiation was conducted with BayPass v2.1 (Gautier, 2015). First, we ran BayPass to estimate the scaled variance–covariance matrix (Ω) under the neutral core model implemented in the software. For both GBS and Rapture datasets, 100 short pilot runs with 1,000 iterations each were set with a 5,000 burn‐in period. We then ran BayPass with the same settings defined for individual‐based data but accounting for the specificities of Pool‐seq data using the “Pool‐seq” options implemented in BayPass. As for *F*_ST_ analysis, only the SNP with the highest MAF at each locus for each dataset was kept. To investigate population structure, we carried out a singular value decomposition on each Ω matrix. We used the resulting principal components coordinates to produce a two‐dimensional visualization of the observed genetic variation. We compared the geographic position (i.e., latitude and longitude) of sampling sites with their PC‐based genetic positions. The correlation between Ω matrices was then assessed using a Mantel test. The Pool‐seq variance–covariance matrix was reduced by averaging over pool site replicates in order to perform this latter analysis with similar matrix sizes as the individual matrix (i.e., individual‐based Ω matrices of size 6 × 6).

## 3 RESULTS

### 3.1 Sequencing data statistics

The average number of reads per sample among sequenced libraries was 1.3 million (*SD* = 0.41), 0.46 million (*SD* = 0.25), and 8.4 million (*SD* = 1.7) for GBS, Rapture, and Pool‐seq, respectively.

The Ion Proton protocol used at the IBIS sequencing platform provides 80 million reads per sequencing for one chip on average. From our Rapture experiment (i.e., multiplexing with 384 sample libraries per Ion Proton chip with two sequencing runs), we expected to retrieve roughly 0.4 million reads per sample (i.e., 2 chips × 80 million reads divided by 384 samples libraries). Conversely, we multiplexed only 96 samples per chip for GBS sequencing, yielding an expected number of 1.6 million reads per sample. Here, it is noteworthy that GBS sequencing represented the entire genome sequence diversity obtained from restriction enzyme libraries, whereas Rapture libraries represent only a reduced fraction of the GBS libraries (i.e., 9,818 captured sequences). Hence, the useful genomic load (i.e., proportion of expected reads that are both present in the reference and in the raw data) between the two protocols would not be the same. From our catalog of reference containing 9,818 loci, the average proportion of targeted loci recovered (with at least one read) was comparable yet slightly lower for Rapture mapped data (95%) compared to GBS and Pool‐seq (98% and 99%, respectively). After filtration, the number of SNPs discovered by GBS and Rapture was 16,986 and 13,931, respectively, while SNPs calling from Pool‐seq discovered a total of 10,874 filtered SNPs (Table 1). Missing threshold removed nine and 27 individuals from GBS and Rapture dataset, respectively. Several samples showing a suspected DNA contamination were also removed from the GBS and Rapture datasets. Final datasets included a total of 265 and 252 individuals for GBS and Rapture, respectively. Complete details of sequencing outputs and data processing results are summarized in Table 1. Finally, selecting only one SNP per locus gave 8,079; 6,401 and 5,558 SNPs for GBS, Rapture, and Pool‐seq datasets. For downstream analyses, the complete SNPs dataset genotyped in each method is called the “overall SNP dataset”. Sequencing information and bioinformatics results about de novo individual‐base catalog from GBS sequencing are also provided in Table S1.

**Table 1:** Summary statistics of data obtained using genotype by sequencing (GBS), Rapture, and Pool‐seq approaches

	GBS	Rapture	Pool‐seq
Number of individual barcodes per sequencing chip	*N* = 96	*N* = 384	*N* = 16 or 6
Average reads per library (millions)	80 (*SD* = 3.3)	84 (*SD* = 4.2)	78 (*SD* = 12.3)
Average reads per individual/pool (millions)	1.3 M (*SD* = 0.41)	0.46 M (*SD* = 0.25)	8.4 M (*SD* = 1.7)
Proportion of targeted loci with at least one read per sample/pool	98%	95%	99%
SNPs called	41,147	35,325	49,238
SNPs quality filtering	16,986	13,930	10,874
SNPs (only one SNP per locus)	8,079	6,401	5,558
SNPs mean depth	17×	33×	87×
% targeted loci after filtering	82%	65%	56%

Note

The last line (% targeted loci after filtering) indicates the proportion of loci kept at the end of the filtering steps and relative to the maximum of loci expected (i.e., the 9,818 loci from the reference catalog used for mapping and for sequence capture).

### 3.2 Consistency of estimated allele frequencies

**Figure 2:**
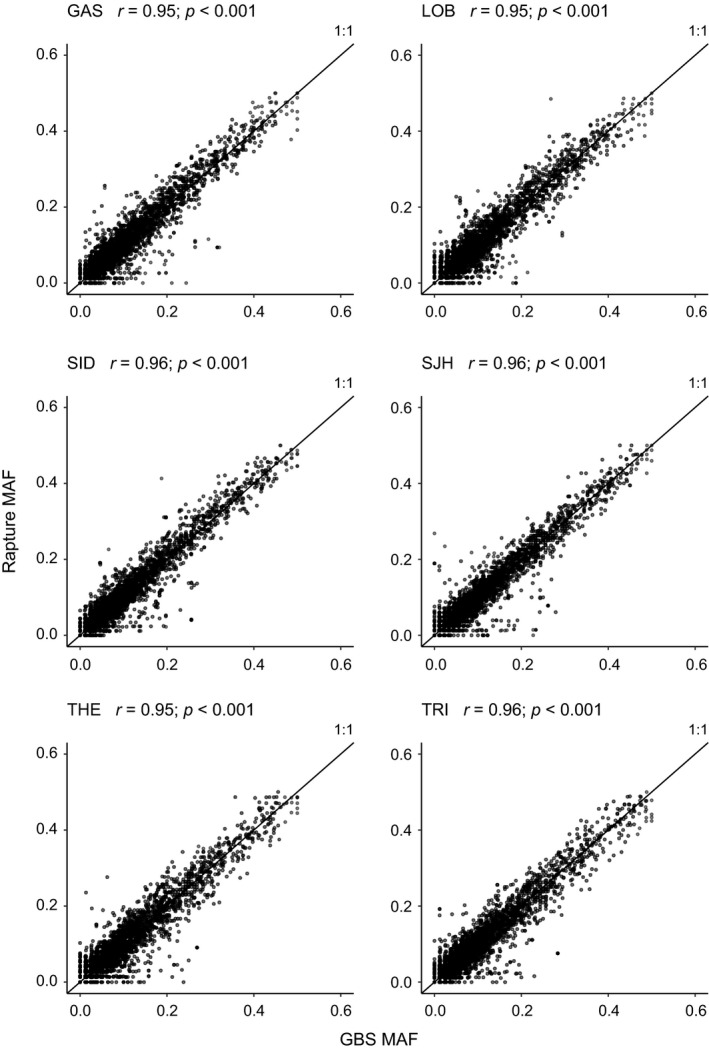
Minor allele frequency correlation comparing GBS and Rapture. Comparison between minor allele frequency (MAF) estimates of the 4,664 overlapped SNPs from individual GBS (*x*‐axis) and Rapture (*y*‐axis), with the Pearson correlation values for each population comparison. The black line represents the expected correlation (1:1 proportion)

**Figure 3:**
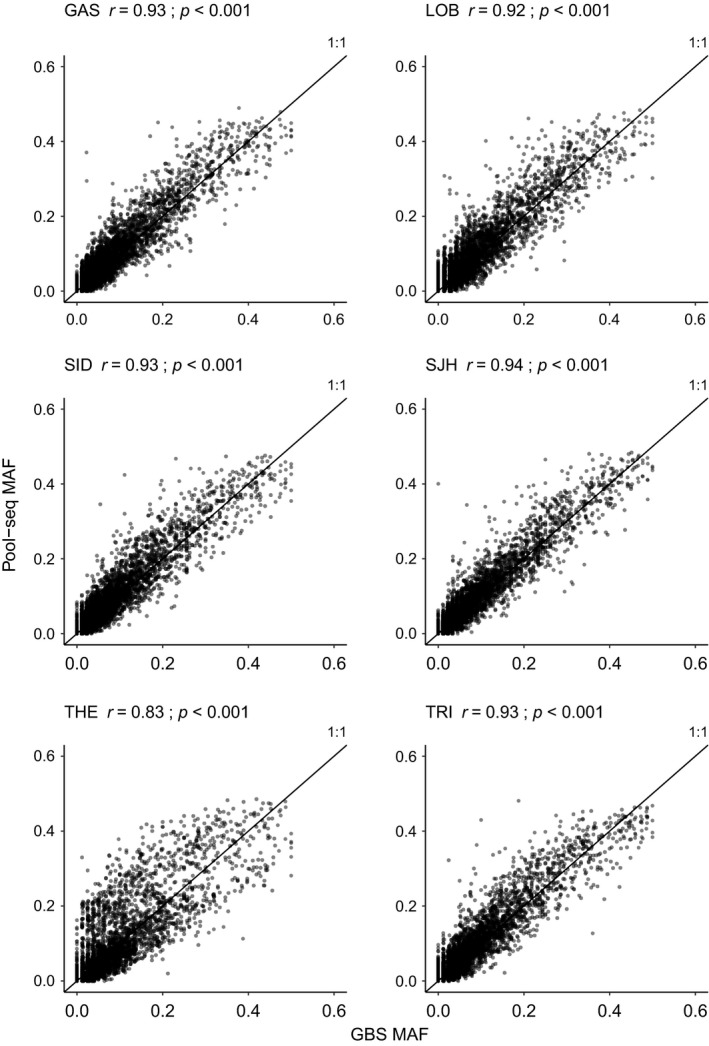
Minor allele frequency correlations comparing GBS and Pool‐seq. Comparison between minor allele frequency (MAF) estimates for the 4,664 overlapping SNPs from individual GBS sequences data (*x*‐axis) and Pool‐seq data (*y*‐axis), with the Pearson correlation values for each comparison. For each sampling site comparison, Pool‐seq values represent the average of minor allele frequency between pool replicates. The black line represents the expected correlation (1:1 proportion)

In total, 4,664 SNPs were shared among the three methods which is referred to “overlapped dataset” hereafter (see Figure S1). Minor allele frequencies were highly correlated among the three methods tested (mean *r* = 0.934, *SE* = 0.035). Pearson correlation between GBS and Rapture allele frequency for a given population was 0.95 on average (Figure 2), while correlation between GBS and Pool‐seq was 0.92 on average and variable among populations (Figure 3, see also Table 2 for each pool replicate). It was clear from the data that the average correlation value for Pool‐seq was driven downward by a lower correlation value observed in one of the three THE replicates (i.e., THE(1), Pearson *r* = 0.64). However, no difference in coverage was observed for THE(1) compared to other pool replicates THE(2) and THE(3) (see Figure S3). Thus, we suspected that individual DNA contributions in THE(1) were strongly unbalanced, probably due to experimental errors when samples DNA were pooled together. Therefore, the pool replicate THE(1) was removed from all Pool‐seq datasets in order to mitigate its effect in downstream analyses (both for overall and overlapped SNPs datasets).

**Table 2:** Details of minor allele frequency (MAF) correlations between individual‐based approaches (i.e., GBS and Rapture) and Pool‐seq overlapped SNPs datasets

	GAS	LOB	SID	SJH	THE	TRI
GBS versus						
Pool replicate 1	0.86	0.85	0.87	0.92	0.64	0.85
Pool replicate 2	0.86	0.88	0.86	0.88	0.84	0.88
Pool replicate 3	0.86	0.85	0.86	0.91	0.86	0.86
Pool replicate 4	0.85	0.80	0.84	–	–	0.87
Average	0.86	0.85	0.86	0.90	0.78	0.87
Rapture versus						
Pool replicate 1	0.84	0.84	0.87	0.89	0.61	0.84
Pool replicate 2	0.85	0.87	0.85	0.86	0.82	0.87
Pool replicate 3	0.85	0.84	0.85	0.88	0.84	0.84
Pool replicate 4	0.85	0.78	0.83	–	–	0.86
Average	0.85	0.83	0.85	0.88	0.76	0.85

Note

Values represent MAF correlations between individual‐based data and each Pool‐seq replicate distributed for each sampling site (columns). Sampling site codes are detailed in the Figure 1 (i.e., sampling map). All correlation values were significant (*p*‐value < 10^−4^) and calculated from the Pearson method. Note the weaker correlation for Pool replicate 1 for the THE population.

### 3.3 Measuring genetic differentiation

Overall, the genetic differentiation measured by the three methods was weak with an average pairwise *F*_ST_ of 0.0028 (*SD* = 0.0027, Table 3). While nearly identical levels of genetic differentiation were observed among individual‐based data (e.g., average *F*_ST_ was 0.0012 and 0.0011 for GBS and Rapture, respectively), values obtained from Pool‐seq data were three to five times higher (average *F*_ST_ was 0.0060 for Pool‐seq). Analyzing the coefficient of variation (*SD*/mean) through pairwise *F*_ST_ bootstraps showed a lower variation for Pool‐seq estimates (average CV was 15% for overall and 11% for overlapped dataset) in comparison with individual‐based methods where a higher level of variation was observed (average CV was 37% for overall and 88% for overlapped for GBS and was 12% for overall and 51% for overlapped for Rapture). The Mantel tests of IBD were significant for all datasets (see Table S2). However, GBS and Rapture displayed stronger correlation (observed Mantel test *r*‐value = 0.70 for the overall and 0.62 for the overlapped for GBS datasets; *p*‐value < 0.05; *r*‐value = 0.82 for the overall and 0.66 for the overlapped for Rapture datasets; *p*‐value < 0.05) than Pool‐seq (Mantel test *r*‐value = 0.45; *p*‐value = 0.0010 for both overall and overlapped Pool‐seq datasets).

**Table 3:** Genetic differentiation (i.e., pairwise *F*_ST_ values) estimated by Weir and Cockerham (1984) index

Samples pair	Overall SNPs datasets			Overlapped SNPs dataset		
	GBS	Rapture	Pool‐seq	GBS	Rapture	Pool‐seq
GAS|LOB	0.0021 [0.0014 to 0.0029]	0.0022 [0.0014 to 0.0029]	0.0077 [0.0066 to 0.0088]	0.0016 [0.0004 to 0.0028]	0.0017 [0.0007 to 0.0027]	0.0081 [0.0068 to 0.0093]
GAS|SID	**0.0001** **[−0.0004 to 0.0008]**	**0.0004** **[−0.0001 to 0.0011]**	0.0046 [0.0036 to 0.0055]	**0** **[−0.0010 to 0.0009]**	**0** **[−0.0011 to 0.0005]**	0.0060 [0.0048 to 0.0071]
GAS|SJH	0.0030 [0.0023 to 0.0037]	0.0018 [0.0011 to 0.0025]	0.0033 [0.0023 to 0.0045]	0.0020 [0.0010 to 0.0031]	0.0015 [0.0005 to 0.0024]	0.0041 [0.0029 to 0.0053]
GAS|THE	0.0026 [0.0018 to 0.0032]	0.0015 [0.0007 to 0.0022]	0.0085 [0.0071 to 0.0100]	0.0013 [0.0003 to 0.0022]	**0.0008** **[−0.0001 to 0.0017]**	0.0095 [0.0081 to 0.0110]
GAS|TRI	0.0006 [2.5e^−5^ to 0.0012]	0.0006 [3e^−5^ to 0.0012]	0.0047 [0.0038 to 0.0056]	**0.0004** **[−5e^−5^ to 0.0013]**	**0.0004** **[−4e^−4^ to 0.0013]**	0.0061 [0.0050 to 0.0073]
LOB|SID	0.0010 [0.0002 to 0.0016]	0.0017 [0.0010 to 0.0024]	0.0071 [0.0060 to 0.0082]	**0.0010** **[−5e^−5^ to 0.0020]**	0.0016 [0.0007 to 0.0026]	0.0078 [0.0066 to 0.0090]
LOB|SJH	0.0009 [0.0002 to 0.0016]	**0** **[−0.0007 to 0.0006]**	0.0030 [0.0019 to 0.0040]	0.0012 [0.0002 to 0.0023]	**0.0007** **[−0.002 to 0.0017]**	0.0037 [0.0025 to 0.0049]
LOB|THE	**0.0006** **[−3.9e^−5^ to 0.0014]**	**0.0002** **[−0.0005 to 0.0009]**	0.0085 [0.0072 to 0.0098]	**0.0009** **[−0.0001 to 0.0020]**	**0.0006** **[−0.0004 to 0.0015]**	0.0086 0.0071 to 0.0100]
LOB|TRI	0.0018 [0.0010 to 0.0024]	0.0023 [0.0015 to 0.0030]	0.0064 [0.0053 to 0.0073]	0.0012 [0.0001 to 0.0024]	0.0025 [0.0013 to 0.0036]	0.0069 [0.0057 to 0.0081]
SID|SJH	0.0025 [0.0018 to 0.0031]	0.0014 [0.0008 to 0.0020]	0.0032 [0.0021 to 0.0043]	0.0022 [0.0012 to 0.0032]	0.0016 [0.007 to 0.0025]	0.0040 [0.0029 to 0.0053]
SID|THE	0.0021 [0.0014 to 0.0028]	0.0013 [0.0006 to 0.0020]	0.0088 [0.0075 to 0.0101]	0.0011 [4e−5 to 0.0021]	**0.0008** **[−0.0001 to 0.0018]**	0.0098 [0.0083 to 0.0113]
SID|TRI	**0.0002** **[−4e^−5^ to 0.0007]**	0.0006 [1e^−6^ to 0.0012]	0.0047 [0.0038 to 0.0057]	**0.0004** **[−0.0004 to 0.0014]**	**0.0004** **[−0.0004 to 0.0013]**	0.0056 [0.0045 to 0.0067]
SJH|THE	**0** **[−0.0008 to 0.0002]**	**0** **[−0.0014 to −1^e−3^]**	**0.0009** **[−0.0002 to 0.0022]**	**3.23e^−6^** **[−0.009 to 0.0010]**	**0.0002** **[−0.0007 to 0.0013]**	0.0037 [0.0023 to 0.0051]
SJH|TRI	0.0024 [0.0017 to 0.0035]	0.0015 [0.0008 to 0.0021]	0.0024 [0.0014 to 0.0035]	0.0019 [0.0009 to 0.0029]	0.0016 [0.0006 to 0.0026]	0.0036 [0.0024 to 0.0048]
THE|TRI	0.0022 [0.0015 to 0.0029]	0.0019 [0.0011 to 0.0027]	0.0077 [0.0065 to 0.0090]	0.0021 [0.0009 to 0.0031]	0.0021 [0.0010 to 0.0031]	0.0099 [0.0085 to 0.0115]
Average *F*_ST_	0.0014	0.0011	0.0054	0.0011	0.0011	0.0065

Note

95% confidence intervals were obtained after 1,000 bootstraps and are provided below *F*_ST_ values. Sampling sites codes are detailed in the Figure 1 (i.e., sampling map). Values in bold were not significant.

**Figure 4:**
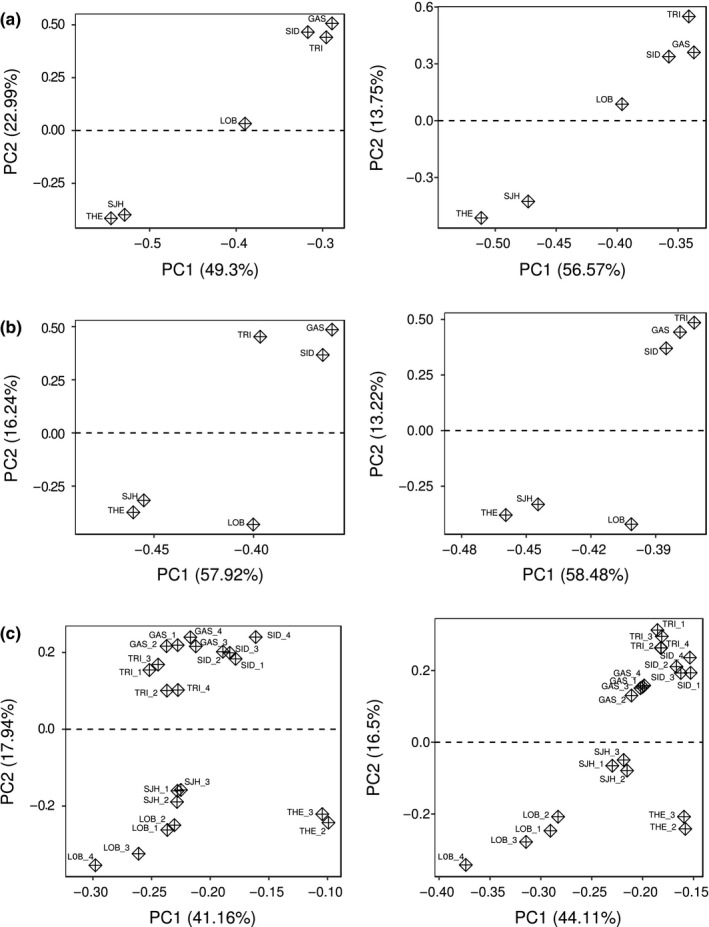
Clustering analysis under Bayesian hierarchical model. (a, b, and c) represent the eigenvalue decomposition of the scaled variance–covariance matrices of population allele frequencies (Ω) for GBS, Rapture, and Pool‐seq datasets, respectively. Left plots correspond to overall SNPs datasets and right plots correspond to overlapping SNPs datasets. Variance–covariance matrix (Ω) was estimated from the neutral core model proposed by Coop, Witonsky, Rienzo, & Pritchard (2010) and implemented in BAYPASS software (Gautier, 2015).

The analysis of the variance–covariance matrices (hereafter Ω matrix) depicted similar pattern of clustering between the three sequencing methods tested. Figure 4 illustrates the resulting principal components coordinates derived from a singular value decomposition of each Ω matrix, calculated for overall and overlapped SNPs datasets. The first axis of variation (PC1) accounted for nearly half of the total genetic variation, ranging from 49.30% to 58.48%, for all three methods. The second axis of variation (PC2) explained 22.54%, 19.76%, and 17.94% for GBS, Rapture, and Pool‐seq methods, respectively, for the overall SNP dataset, while for the overlapped SNP dataset, PC2 explained only 14.09%, 14.17%, and 16.50% of the variance for GBS, Rapture, and Pool‐seq, respectively. The GBS datasets revealed two clusters corresponding to the North and South genetic groups defined by Benestan et al. (2015), although the LOB sampling site was somewhat at an intermediate position along PC1 and PC2 axes (Figure 4a). Rapture and Pool‐seq visual representation of Ω matrices (hereafter Ω‐PC) showed a clear clustering pattern only for the second principal component (Figure 4b,c). Yet, Rapture and Pool‐seq Ω‐PC also depicted the same expected North/South clustering but showed that LOB sample was more closely related to the South group relatively to PC1 and PC2 axes. The correlation between the genetic positions obtained for PC1 and PC2 (Ω‐PC1 and Ω‐PC2), and spatial distribution of sample sites (i.e., latitude and longitude) revealed a significant spatial structure (Table 4). Strong positive correlations were measured for all methods and datasets except for Pool‐seq overall dataset where the correlation on the PC1 was negative (*r* = −0.08 for PC1 vs. latitude, and *r* = −0.15 for PC1 vs. longitude). On average among all datasets, correlation level between Ω‐PC space and the spatial distribution of samples sites was stronger for PC2 (average *r* = 0.50 for Ω‐PC1 vs. spatial distribution, and average *r* = 0.80 for Ω‐PC2 vs. spatial distribution).

**Table 4:** Two‐dimensional association of genetic variation versus geography

	Latitude		Longitude	
	PC1	PC2	PC1	PC2
GBS overall	0.71	0.74	0.69	0.70
GBS overlap	0.69	0.72	0.68	0.78
Rapture overall	0.55	0.94	0.48	0.63
Rapture overlap	0.68	0.94	0.720	0.82
Pool‐seq overall	−0.08	0.87	−0.15	0.76
Pool‐seq overlap	0.60	0.92	0.460	0.79

Note

Values represent Pearson *r* correlation between Ω‐PC space coordinates of each sampling site (i.e., PC1 and PC2, see Figure 4) versus geographic position (i.e., latitude and longitude).

Mantel tests detected positive and significant correlation among all Ω matrices (average *r* = 0.76; Table S3), but stronger correlation coefficients were obtained between Ω matrices from the two individual‐based methods (mean *r* = 0.87), while correlations between either GBS or Rapture Ω matrices and Pool‐seq Ω matrices were lower (mean *r* = 0.67). Correlations between overall versus overlapped datasets among each method tested were still high and significant (*r* = 0.91 for GBS, *r* = 0.97 for Rapture, and *r* = 0.84 for Pool‐seq). This indicates that subsampling overlapped SNPs from overall SNPs dataset generally conserved the genetic relationships among each pair of populations.

## 4 DISCUSSION

Cost‐effective NGS alternatives (i.e., Pool‐seq and Rapture) to conventional individual GBS libraries are becoming increasingly popular and may represent a well‐suited approach for the analysis of genetic variation in wide natural populations. These alternative approaches appear to represent interesting SNP genotyping strategies in the case of species exhibiting weak genetic differentiation, where large sampling design (e.g., extended species range, high number of sampling locations, large sample size, large number of markers) will result in further benefits to accurately investigate genetic structure and connectivity (Gagnaire et al., 2015; Lotterhos & Whitlock, 2015; Patterson et al., 2006).

Here, we empirically explored the consistency of the genetic structure observed in a high gene flow species by comparing conventional GBS with Rapture and Pool‐seq approaches. We found that individual‐based methods (i.e., GBS and Rapture) provided more congruent results than Pool‐seq. In the following sections, we discussed the consistency of these three methods in a context of a weak genetic differentiation and we also highlight the cost and benefits for each method tested.

### 4.1 Level of congruence between GBS and alternatives methods

Our results showed that allele frequencies estimated from GBS and the two alternatives methods, Rapture and Pool‐seq, were consistent although allele frequency estimates from Rapture were more highly correlated to GBS than Pool‐seq. These observations are in agreement with other studies that also reported a strong correlation between pooled and individually measured allele frequencies (Bélanger, Esteves, Clermont, Jean, & Belzile, 2016; Fracassetti et al., 2015; Gautier et al., 2013; Rellstab, Zoller, Tedder, Gugerli & Fischer, 2013). Levels of genetic differentiation among the three sequencing approaches were weak (e.g., all pairwise *F*_ST_ were well under 0.01, Table 3), as often the case for marine species (Gagnaire et al., 2015; Hedgecock, Barber, & Edmands, 2007; Palumbi, 2003). Average *F*_ST_ observed for GBS and Rapture were almost identical and very similar to the level of genetic differentiation previously reported on this species for a larger set of samples with an averaged *F*_ST_ value of 0.0018 across 10,156 SNPs (Benestan et al., 2015).

In contrast, *F*_ST_ estimation from Pool‐seq data was five times higher than those measured with individual‐based methods. However, Pool‐seq *F*_ST_ measures generated lower coefficient of variation through bootstrapping across loci than GBS and Rapture. Lower coefficient of variation for Pool‐seq is likely due to the fact that both allele frequencies and pairwise *F*_ST_ values from Pool‐seq data were actually based on the average estimated for several Pool‐seq replicates, which may have contributed to reduce variance between replicates. This was recommended by previous studies in order to mitigate bias in estimations of allele frequencies potentially caused by the unbalanced contribution of each individual in a pool. Indeed, balancing the DNA contribution of each sample in a pool is notoriously challenging (i.e., equimolarity, Futschik & Schlötterer, 2010; Gautier et al., 2013). Yet, the pool sample size is a critical parameter for characterizing genetic structure from Pool‐seq data, particularly for *F*_ST_ estimation since numerous computational approaches, such as maximum likelihood estimates (Leblois et al., 2018; Smadja et al., 2012) or model‐based methods (Fariello et al., 2017), are conditioned by sample size. In practice, unequal contributions of each individual to the final pool of sequences may introduce biases in allele frequencies estimates (Gautier et al., 2013; Zhu, Bergland, González, & Petrov, 2012). The concept of effective pool size (i.e., number of diploid individuals with equimolar amounts of DNA in an idealized pool that was expected to show the same level of variance in allele frequency estimations) has been proposed by Gautier et al. (2013) to illustrate this latter source of errors. Using an empirical dataset, they showed that the effective pool size could be up to 30% lower than the experimental pool size. Here, using the program *poolne_estim* developed by Gautier et al. (2013), we estimated that the effective pool size ranged from 17 to 48 among all pool replicates (see details in Table S4). This latter results represented an experimental error (as defined by Gautier et al. (2013)) ranging from 0% to 133.6% (average = 55.1%, *SD* = 29.2). Thus, like Gautier et al. (2013), we observed that the effective pool size differed from our experimental pool design. Nevertheless, allele frequency estimates remained similar between individual‐based methods and Pool‐seq, except for one pool. Unfortunately, Gautier et al. (2013) did not explore the impacts of effective pool size on summary statistics such as *F*_ST_ estimates.

Here, we used the *F*_ST_ estimator of Hivert et al. (2018) which apparently outperform earlier proposed estimators (i.e., Popoolation2—Kofler, Pandey, et al., 2011b). Using simulated data, the authors compared the accuracy and robustness of their *F*_ST_ estimator under several sources of bias that commonly affect sequencing datasets. They demonstrated that their estimator was robust regarding the variance of coverage across loci and observed that sequencing error implicated a negligible bias for Pool‐seq *F*_ST_ estimates. However, they found that experimental errors have a substantial effect on *F*_ST_ estimates and represented the most important source of bias in *F*_ST_ estimates between Pool‐seq versus individual‐based genotyping methods. They also noted that the smaller is the pool size, the higher is the effect of experimental bias. For example, they showed that, with an experimental error of 50% and a pool size of *n* = 10, *F*_ST_ estimates were biased by a factor of 1.5. This bias was clearly flattened when the pool size was increased to *n* = 100. In our case, we pooled 48 individuals to create each pool replicate. According to the observations reported by Hivert et al. (2018) and our experimental error estimates, we expect the experimental error (i.e., the effective pool size vs. experimental pool size) to be the main source of bias explaining the difference in *F*_ST_ estimates between individual‐based data and Pool‐seq data. Beyond this bias, slight differences in the *F*_ST_ equation used for individual data and pool‐seq data may also contribute to the empirically observed *F*_ST_ differences. However, Hivert et al. (2018) found that this bias is extremely low (bias < 0.5%). Indeed, their *F*_ST_ calculation model assumes that the read counts are multinomially distributed and suppose that each SNPs have equal sequencing coverage among samples in a pool.

Finally, we were not able to quantify the accuracy of each method in estimating allele frequencies (and derived summary statistics) since we used a purely empirical data in which the truth is unknown. A thorough a simulation study would be relevant to compare the three protocols and complement our outcomes. While simulation studies of Pool‐seq and GBS data have already been performed to report sources of bias (e.g., Arnold, Corbett‐Detig, Hartl, & Bomblies, 2013; Cariou, Duret, & Charlat, 2016; Gautier et al., 2013; Guo et al., 2013; Hivert et al., 2018), no study pertaining to the limits of the Rapture method has been performed yet. Further simulations considering the Pool‐seq, Rapture, and GBS methodologies would allow testing the effect of different levels of pooling, different number of individuals and different number of SNPs on the accuracy of *F*_ST_ estimates for these three libraries protocols. This would enable providing detailed guidelines for designing future empirical studies. However, such work was beyond the goal of the present study that only took an empirical approach to measure consistency among methods. Moreover, as stipulated by Shafer et al. (2017), it is difficult to reproduce the important variation introduced during wet laboratory data generation using simulated data. Indeed, building proper algorithms simulating complex laboratory biases such as PCR duplicates remains difficult as well as similar statistic estimators for all sequencing methods. Hence, we think that our empirical data can still be a relevant approach to substantiate interpretations and test the consistency of the alternatives methods to GBS.

NGS approaches have some conceptual and methodological limitations that can introduce artifacts and affect estimates of population genetic parameters (Andrews et al., 2016; Cariou et al., 2016; Davey et al., 2011). For example, mutations in restriction sites, referred to allele dropout (“ADO”), may result in an underestimation of genetic diversity and false inference of population divergence (Arnold et al., 2013; Cariou et al., 2016; Gautier et al., 2013). Moreover, restricted digested libraries can suffer from a high level of sequence clonality related to PCR amplification (i.e., PCR duplicates), which have the potential to bias allelic read depth and produce genotyping errors (Davey et al., 2011). These methodological limitations are known to generate missing data that can substantially cause mis‐estimations of commonly used statistics (e.g., *F*_ST_, Tajima's D, nucleotide diversity) as well as bias in population genomic inferences (Arnold et al., 2013). In this study, we used Ion Proton™ systems, which were designed to produce single‐end sequencing reads. However, unlike paired‐end sequences data, distinguishing PCR duplicates in single‐end sequences remains difficult. To date, only one recent study provided estimate of the average PCR duplication rate of single‐end high‐throughput sequences datasets (Bansal, 2017). Moreover, the identification of PCR duplicates is difficult in single‐end Pool‐seq since haplotype information is lost. Still, we mitigated potential experimental bias due to PCR duplication by maximizing genomic diversity in each DNA library (i.e., number of genomes), using 200 ng of genomic DNA per sample as recommended by several studies (Andrews et al., 2016; Casbon, Osborne, Brenner, & Lichtenstein, 2011; Davey et al., 2011). Considering that the genome size of the American lobster was estimated roughly 4.5Gb (Jimenez, Kinsey, Dillaman, & Kapraun, 2010), we expected that each DNA library was represented by nearly 40,000 American lobster genomes following the equation:

$$\text{Number}\;\text{of}\;\mathrm{copies}=\frac{Q_{\mathrm{ng}}\cdot 6.022\cdot 10_{\mathrm{molecules}/\mathrm{mole}}^{23}}{G_{\mathrm{bp}}\cdot 650_{g/\mathrm{mole}}\cdot 10_{g/g}^{9}}$$

where *Q*_ng_ is the amount of DNA in nanograms and *G*_bp_ is the length of DNA amplicon in base pairs (i.e., genome size). This calculation is based on the assumption that the average weight of base pair (bp) is 650 Daltons. We also used a sequence size selection (BluePippin™ prep‐Sage Science) to minimize amplicon size variability and limit PCR cycling to 10 cycles during library preparation, two measures that should prevent efficiently the formation of PCR duplicates. Finally, the probability of obtaining PCR duplicates is negatively correlated to the number of targeted markers. Therefore, Ali et al. (2016) targeted 500 loci and observed a high proportion of PCR duplicates. Here, on the contrary, we targeted 9,818 loci, resulting in lower probability of generating PCR duplicates.

### 4.2 Consistency of population structure patterns

Population genetic structure was further investigated using a hierarchical Bayesian model available in BayPass (Gautier, 2015). All the three methods showed a similar signal of clustering, uncovering the presence of two geospatial groups (Figure 4). This North/South dichotomy was previously highlighted using 13 microsatellites (Kenchington, Harding, Jones, & Prodöhl, 2009) and 10,156 SNPs (Benestan et al., 2015), which then give support to our outcomes. Nevertheless, caution is required to interpret this clustering signal. Indeed, comparing observed genetic variation from Ω matrices with spatial distribution of samples sites (i.e., latitude and longitude) revealed that the latitude criterion was the most prominent pattern of clustering. Furthermore, comparing genetic differentiation (i.e., pairwise *F*_ST_) with geographic distances depicted a positive signal of IBD, as also identified by Benestan et al. (2015). Importantly, the disjointed range of sampling sites (i.e., geographic gap between samples from the North vs. South) may impact our capability to accurately resolve population structure (Bradburd, Coop, & Ralph, 2018). Indeed, most currently available clustering methods are known to be easily confounded by the presence of IBD and tend to split continuous patterns of spatial variations in discrete groups (Frantz, Cellina, & Krier, 2009; Meirmans, 2012). Adding intermediate sampling points and using recently developed methods able to deal with both clusters and continuous variation (Bradburd et al., 2018) could help to discriminate the scale of the clustering pattern as well as the pattern of IBD.

### 4.3 Cost considerations of alternatives methods

**Figure 5:**
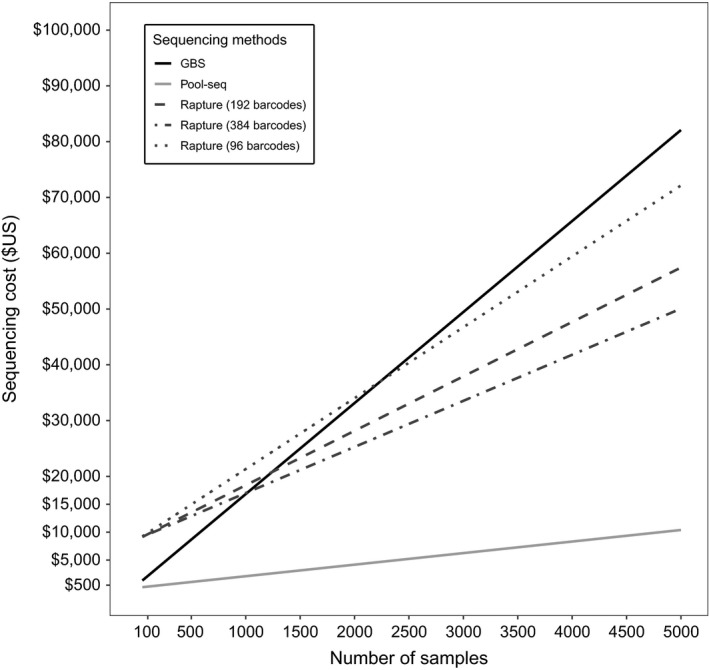
Genotyping cost relatively to sampling design. Genotyping costs were estimated from our experimental design and sequencing platform fees. *Genotyping by sequencing* (GBS) was based on 96 barcodes sequencing setup. Pool‐seq genotyping costs were calculated based on pool size with 50 samples, three technical replicates per pool, and 15 Pool‐seq libraries per sequencing chip. Rapture costs are given for three multiplexing scenarios (e.g., 96, 192, and 384 individual barcodes). Genotyping cost were estimated based on Probe kit invest (here 20K probes kit ≈ 6,000 $US—Arbor Biosciences™ 2016), an average reads depth to 15×, and an optimized capturing step for five Rapture in the same laboratory experience. We also allowed 10% of poor‐quality samples for re‐sequencing in GBS and Rapture. We fixed two sequencing runs for each individual/pool libraries among each approach

GBS, Pool‐seq, and Rapture approaches did not require the same investment of cost and time. For instance, individual‐based approaches such as GBS and Rapture require separating each individual sample when preparing libraries, whereas Pool‐seq preparation involves a single library step for an entire pool of samples (Schlötterer et al., 2014). NGS library preparation still stands as a key cost factor in population genomic studies. So far, Pool‐seq has remained the most economical method to reduce sequencing costs over a large sampling design. However, Rapture may overcome some of the current limitations of Pool‐seq at a reasonable price when using large number of samples. The Figure 5 illustrates the estimated cost of each method relative to the number of samples analyzed and shows that Rapture cost‐effective performance was strongly related to the level of multiplexing. Importantly, we estimated that genotyping costs were comparable between GBS and Rapture—considering Rapture 384 multiplexing setup—when a total of about 1,000 samples are used. Below this threshold, GBS approach remains less expensive than Rapture. Indeed, conversely to GBS, Rapture requires an investment prior to sequencing, in order to select target DNA sequences and then the purchase of capture probes. We estimated that this investment represented near 50% of the total budget for 1,000 samples (following our experiment parameters). On the other hand, Rapture sequencing costs were 22.7% lower relative to GBS (25,390 $US vs. 32,815 $US) based on 2,000 samples, and 40% lower with 5,000 samples (50,445 $US vs. 82,524 $US). Note that GBS sequencing costs were estimated from our experiment (i.e., 96 barcodes setup per sequencing chip with Ion Proton technology) based on the most common protocol implemented by the sequencing platform (IBIS, Canada). Thus, cost saving is achieved by increasing the number of samples per sequencing effort regardless of the sequencing technology (e.g., Ion Proton or Illumina). Furthermore, GBS and Pool‐seq differ from Rapture in terms of time required to produce the final data. Here, we have roughly estimated that for a sample of size 1,000, both GBS and Pool‐seq required approximately nine months, whereas Rapture required near that 16 months due to the initial development steps (see time‐stepping scheme in Figure S2). Consequently, it may be unnecessary and more expensive to use Rapture compared to conventional GBS depending on the scientific question, the scale of the research project, the sampling design, and the laboratory possibilities.

Table 5 provides a summary of prominent advantages and disadvantages of each method that are briefly summarized below. The advantage of GBS approach is that it can be applied using a de novo assembled catalog (Etter, Preston, & Bassham, 2011), while both Rapture and Pool‐seq approach require prior genomic reference in order to align sequencing reads (Ali et al., 2016; Schlötterer et al., 2014). Since no reference genome was available for the American lobster (or even for a closely related organism), we used prior GBS development to select a set of SNP markers and then build a de novo reference catalog, which was used to align raw reads obtained from each approach (e.g., GBS, Rapture, and Pool‐seq). While Ali et al. (2016) used a reference genome for mapping, we demonstrated here that Rapture approach may provide good quality data successfully obtained from a de novo reference catalog based on GBS short reads data.

**Table 5:** Advantages/disadvantages of each approaches

Method	Term definition	Advantage	Disadvantage
GBS	*Introduced by*Davey et al. (2011) Genotyping‐by‐sequencing (GBS) is a set of genetic screening techniques using restriction enzymes to reduce genome complexity and enable high‐throughput genotyping of multiple DNA samples at large number of DNA marker (usually SNPs)	Keep individual information No reference genome required Allow low coverage sequencing Library normalization	High genotyping costs with large number of samples^a^ Heavy bioinformatics process when dealing with thousands of samples Limited multiplexing for sequencing^b^
Rapture	*Developed by*Ali et al. (2016) “A sequencing technique, which combine the benefits of both RAD‐seq and sequence capture adding an in‐solution capture of chosen RAD tags to target sequencing reads to desired loci. Rapture is a rapid and flexible technology capable of analyzing a very large number of samples with minimal sequencing and library preparation costs.”	Costs decrease with number of samples compared to GBS Keep individual information No reference genome required Allow low coverage sequencing Fast bioinformatic processes Requires fewer reads per sample than GBS for the same coverage	Require prior RAD‐seq experiment to develop capture probes Investment for probes production Overall time required for getting results extended Less cost‐effective when number of samples is small
Pool‐seq	*Reviewed by*Schlötterer et al. (2014) “A sequencing technique in which sequencing libraries are not prepared from DNA of a single individual or cell but from a mixture of DNA fragments originating from different individuals or cells.”	Low costs Fast library time preparation Large library multiplexing (hundreds to thousands of samples) Fast bioinformatics processes	No individual information Requires genomic reference Require pool of individuals > 40 Unbalanced contribution of samples Minimal coverage > 20×

a Genotyping costs are proportional to the number of samples.

b For the same sequencing depth, GBS need more sequencing effort per sample than Rapture.

## 5 CONCLUSION

In conclusion, we found that Pool‐seq and Rapture provided consistent allele frequency estimates and nearly identical patterns of population structure compared to conventional GBS approach. However, despite increasingly accurate *F*_ST_ estimator for Pool‐seq methods (Hivert et al., 2018), or the availability of individual data combined with higher sequencing depth for the Rapture method, we found that estimating very weak genetic differentiation in empirical data remains difficult no matter the genotyping method being used. We flagged up the importance of unequal contribution of samples in Pool‐seq that introduce substantial bias in *F*_ST_estimates. Therefore, increasing the size of the pools (i.e., over 100 samples per pool) may help to further reduce the effect of this experimental bias. We further advocate that future empirical Pool‐seq projects would be reinforced with several pool replicates in order to control for experiment reproducibility and data robustness.

## CONFLICT OF INTEREST

None declared.

## AUTHOR CONTRIBUTIONS

L. Bernatchez and R. Rochette designed and supervised the project. L. Benestan conducted the field sampling. Y. Dorant conducted genomic data analyses and lead on writing the manuscript with contributions from L. Benestan and Q. Rougemont. All authors contributed to analyses and interpretation of the results in their respective expertise and approved the final version of the manuscript.

## Data Availability

DNA sequences (FASTQ format) for each approach (i.e., GBS, Rapture, and Pool‐seq) demultiplexed over individual/pool samples at: NCBI. The following datasets generated for this study and available from the Dryad Digital Repository: https://doi.org/10.5061/dryad.64f7982
GBS VCF file of no filtered SNPs dataset. Rapture VCF file of no filtered SNPs dataset. Pool‐seq synchronized file (Popoolation2). Whitelist of overlapped SNPs. Reference catalog.
