# Supplementary material for: Comparing Pool‐seq, Rapture, and GBS genotyping for inferring weak population structure: The American lobster (Homarus americanus) as a case study
Source: Ecol Evol. 2019 May 26;9(11):6606–23. doi: 10.1002/ece3.5240 (PMC6580275; doi:10.1002/ece3.5240)
Supplement: Supplementary file 1 [file ECE3-9-6606-s001.zip › ece35240-sup-0001-AppendixS1/ece35240-sup-0001-TableS1.docx]

| Number of individuals | 192 |
| --- | --- |
| Total number of single-end reads (millions) | 327 |
| Mean number of reads per sample (millions) | 1.3 (sd=0.36) |
|  |  |
| SNP discovery from *de novo* Stacks catalog (v.1.38) | SNP count |
| Stacks catalog – Putative loci | 96,110 |
|  |  |
| **SNPs filtering steps*** |  |
| SNP presence > 70% in each sampling site | 50,174 |
| H_obs_ < 0.6 | 908 |
| FIS between [-0.7 : 0.7] | 14,979 |
| Minor Allele Frequency threshold > 1% in global | 38,945 |
| Minor Allele Frequency threshold > 5% in each sampling site | 59,513 |
| Maximum of 8 SNPs allowed per locus | 2,621 |
| Highly similar sequences filter | 227 |
|  |  |
| **Final SNPs *de novo* catalog for alignment reference** |  |
| Number of putative SNPs | 16,780 |
| Number of 80bp sequences as putative loci** | 9,818 |
