# Supplementary material for: Comparing Pool‐seq, Rapture, and GBS genotyping for inferring weak population structure: The American lobster (Homarus americanus) as a case study
Source: Ecol Evol. 2019 May 26;9(11):6606–23. doi: 10.1002/ece3.5240 (PMC6580275; doi:10.1002/ece3.5240)
Supplement: Supplementary file 1 [file ECE3-9-6606-s001.zip › ece35240-sup-0001-AppendixS1/ece35240-sup-0002-TableS2.docx]

| Matrix A | Matrix B | Mantel’s r | P-value |
| --- | --- | --- | --- |
| Genetic GBS^a^ | Sea floor distances | 0.706 | 0.025 |
| Genetic GBS^b^ | Sea floor distances | 0.620 | 0.034 |
| Genetic Rapture^a^ | Sea floor distances | 0.822 | 0.012 |
| Genetic Rapture^b^ | Sea floor distances | 0.659 | 0.025 |
| Genetic Pool-seq^a^ | Sea floor distances | 0.453 | 0.001 |
| Genetic Pool-seq^b^ | Sea floor distances | 0.456 | 0.001 |
