# Supplementary material for: Comparing Pool‐seq, Rapture, and GBS genotyping for inferring weak population structure: The American lobster (Homarus americanus) as a case study
Source: Ecol Evol. 2019 May 26;9(11):6606–23. doi: 10.1002/ece3.5240 (PMC6580275; doi:10.1002/ece3.5240)
Supplement: Supplementary file 1 [file ECE3-9-6606-s001.zip › ece35240-sup-0001-AppendixS1/ece35240-sup-0003-TableS3.docx]

|  | GBS^a^ | GBS^b^ | Rapture^a^ | Rapture^b^ | Pool-seq^a^ | Pool-seq^b^ |
| --- | --- | --- | --- | --- | --- | --- |
| GBS^a^ | - | 0.001 | 0.001 | 0.001 | 0.014 | 0.013 |
| GBS^b^ | 0.91 | - | 0.006 | 0.002 | 0.016 | 0.012 |
| Rapture^a^ | 0.88 | 0.80 | - | 0.005 | 0.025 | 0.017 |
| Rapture^b^ | 0.86 | 0.81 | 0.97 | - | 0.024 | 0.033 |
| Pool-seq^a^ | 0.75 | 0.67 | 0.69 | 0.63 | - | 0.005 |
| Pool-seq^b^ | 0.66 | 0.70 | 0.63 | 0.63 | 0.85 | - |
