# Supplementary material for: Comparing Pool‐seq, Rapture, and GBS genotyping for inferring weak population structure: The American lobster (Homarus americanus) as a case study
Source: Ecol Evol. 2019 May 26;9(11):6606–23. doi: 10.1002/ece3.5240 (PMC6580275; doi:10.1002/ece3.5240)
Supplement: Supplementary file 1 [file ECE3-9-6606-s001.zip › ece35240-sup-0001-AppendixS1/ece35240-sup-0004-TableS4.docx]

| Pool name | *n_e_* posterior mean | Experimental error in % |
| --- | --- | --- |
| GAS_1 | 29.2 (1.49) | 80.3 (5.2) |
| GAS_2 | 29.7 (1.47) | 78.6 (5.1) |
| GAS_3 | 32.2 (1.85) | 70.0 (6.1) |
| GAS_4 | 27.5 (1.45) | 86.2 (5.3) |
| LOB_1 | 31.7 (1.58) | 71.6 (5.3) |
| LOB_2 | 47 (0.20) | 3.0 (2.2) |
| LOB_3 | 28.6 (1.47) | 82.3 (5.2) |
| LOB_4 | 17.2 (0.84) | 133.6 (5.1) |
| SID_1 | 37.2 (2.12) | 53.8 (6.9) |
| SID_2 | 31.0 (1.70) | 74.1 (5.8) |
| SID_3 | 30.8 (1.56) | 74.6 (5.3) |
| SID_4 | 25.0 (1.25) | 96.0 (5.0) |
| SJH_1 | 48.0 (0) | 0 (0) |
| SJH_2 | 44.8 (2.45) | 23.5 (13.8) |
| SJH_3 | 48.0 (0) | 0 (0) |
| THE_1 | 45.3 (2.83) | 19.1 (16.5) |
| THE_2 | 47.0 (1.82) | 7.6 (12.6) |
| TRI_1 | 26.9 (1.32) | 88 (4.9) |
| TRI_2 | 47.2 (1.36) | 7.5 (10.8) |
| TRI_3 | 26.8 (1.37) | 88.8 (5.1) |
| TRI_4 | 45.7 (2.10) | 18.6 (13.3) |
