# Supplementary material for: Comparing Pool‐seq, Rapture, and GBS genotyping for inferring weak population structure: The American lobster (Homarus americanus) as a case study
Source: Ecol Evol. 2019 May 26;9(11):6606–23. doi: 10.1002/ece3.5240 (PMC6580275; doi:10.1002/ece3.5240)
Supplement: Supplementary file 1 [file ECE3-9-6606-s001.zip › ece35240-sup-0001-AppendixS1/ece35240-sup-0008-AppendixS1.docx]

**Appendix S1**

**Table S1: Sequencing and filtration details of the *de novo* individual-based GBS catalog**

Note : This *de novo* catalog of filtered SNPs was used as reference catalog for GBS, Rapture, and Pool-seq sequences alignment.

* Number of putative SNPs that failed to pass filters. Note that the same putative SNP could fail for multiples filters.
** Note that these 9,818 targeted loci were used to design Rapture probes.

**Table S2 : Results of standard Mantel tests between the genetic and geographic distances**

Genetic distances represented the standardized F_ST_ (*i.e.*, F_ST_ / 1 – F_ST_). Geographic distances corresponded to sea floor distances (*i.e.*, least cost distance matrix) computed through the R package *marmap* 0.9.6 (Pante & Simon-Bouhet, 2013). Mantel tests were performed using 1,000 permutations, assuming a two-dimensional habitat in which geographic distance were log-transformed. The a and b symbols represent overall SNPs datasets and overlapped SNPs datasets, respectively.

**Table S3: Mantel test results comparing different Ω matrices.**

Mantel test correlation coefficient (below diagonal) and level of significance are represented by p-values (above diagonal) following 10,000 resampling iterations. The a and b symbols represent overall SNPs datasets and overlapped SNPs datasets, respectively.

**Table S4: Estimation of effective pool size (*ne*) and experimental error among Pool-seq replicates**

For each pool, the experimental pool size was 48 samples. Effective pool size and experimental error were performed using the program *poolne_estim* developed by Gautier *et al.* (2013). Numbers in parenthesis represent the standard deviation.

**Figure S1: Venn diagram of overlapping SNPs between GBS, Rapture, and Pool-seq.**

**The number in brackets refers to the total number of filtered SNPs discovered for each method.**

**Figure S2:** **Estimated time-stepping scheme for each method**

For each method, we estimated the relative time required from DNA extraction (here with a scenario of 1,000 samples) to the bioinformatic analysis (*i.e.,* only steps of SNPs calling). The different steps are represented by letters as follows: **(a)** DNA extraction and normalization of 1,000 samples, **(a*)** DNA extraction and normalization of a samples subset for Rapture probes design, **(b)** libraries preparation and sequencing step, **(c)** bioinformatic analysis (i.e., SNP calling), **(d)** bioinformatic analysis with probes design, and **(e)** Rapture probes synthesis. Note that we supposed an available genomic reference for Pool-seq method.

**Exploring artefacts of Allele frequencies estimates from Pool-seq sampling site THE.**

*Bias of allele frequencies estimates from THE sampling site in Pool-seq.*

Pool-seq experimental design is fully detailed in the Material and Methods section. While all Pool-seq replicates showed an average correlation level of allele frequencies with GBS data over 85%, the Pool replicate THE_1_ showed biased estimates of allele frequencies driving correlation level to 64% (Table 2). To explore this bias, we tested the effect of SNP coverage with the absolute allele frequency difference (hereafter Δ_AF_) between GBS and Pool-seq data (Figure A3, below). We supposed that, under assumption of equal DNA contribution of each sample in the pool, if biased alleles frequencies estimates were affected by abnormal coverage, we will expect to observed either (i) proportion of higher ΔAF with low coverage values, meaning poor library preparation for this pool, or on the other hand (ii) proportion of higher ΔAF with strong coverage values, meaning potential effect of PCR duplicates or sequences paralogs. However, none of the coverage effect was observed when we compared THE_1_ with THE_2_ and THE_3_ (Figure A3). Therefore, high level of noise observed in allele frequencies estimates from THE_1_ could be explain though potential experimental error, namely unequal DNA contribution of each individual in this pool. This was the largest source of experimental error that could explain differences between pooled replicates libraries (Zu *et al.* 2012, Gautier *et al.* 2013).

**References**

Gautier, M., Foucaud, J., Gharbi, K., Cézard, T., Galan, M., Loiseau, A., Thomson, M., Pudlo, P., Kerdelhué, C., Estoup, A. (2013). Estimation of population allele frequencies from next- generation sequencing data: pool-versus individual-based genotyping. *Molecular Ecology*, *22*(14), 3766–3779.

Zhu Y, Bergland AO, González J, Petrov DA (2012) Empirical Validation of Pooled Whole Genome Population Re-Sequencing in Drosophila melanogaster. *PLOS ONE*, 7, e41901.

**Figure S3: Effect of Pool-seq SNP coverage on the absolute allele frequency difference**

Y-axis represents the absolute allele frequency difference ( Δ_AF_ ) between GBS and Pool-seq alleles frequencies estimates. X-axis represents SNP coverage of Pool-seq data. From left to right, Pool replicates THE_1_, THE_2_, and THE_3,_ respectively.
