# Supplementary figures and images for: Comparing Pool‐seq, Rapture, and GBS genotyping for inferring weak population structure: The American lobster (Homarus americanus) as a case study
Source: Ecol Evol. 2019 May 26;9(11):6606–23. doi: 10.1002/ece3.5240 (PMC6580275; doi:10.1002/ece3.5240)

**GBS**

**POOLSEQ**

3852

2316

3315

4664

6154

579

2534

**RAPTURE**

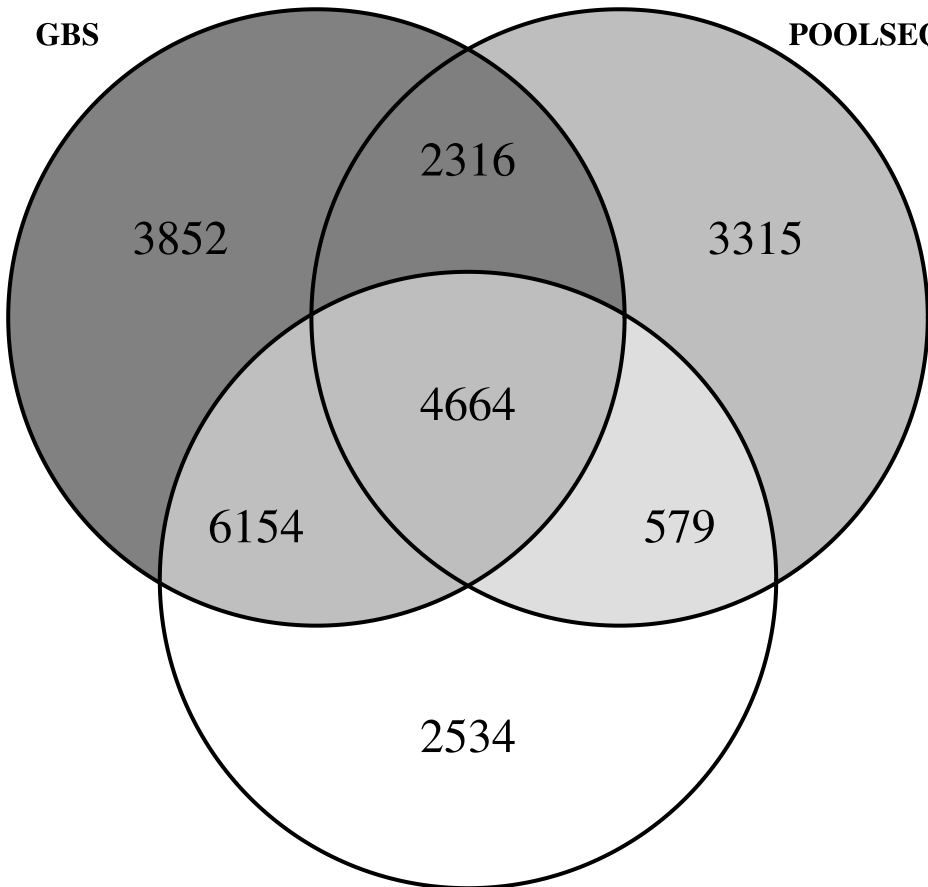

Supplement: Supplementary file 1 [file ECE3-9-6606-s001.zip › ece35240-sup-0001-AppendixS1/ece35240-sup-0005-FigS1.pdf]

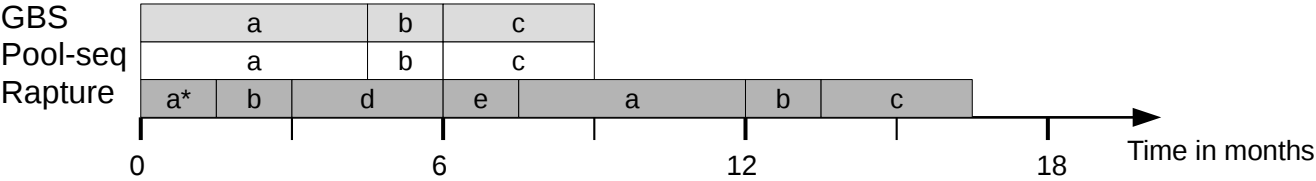

Supplement: Supplementary file 1 [file ECE3-9-6606-s001.zip › ece35240-sup-0001-AppendixS1/ece35240-sup-0006-FigS2.pdf]

$\Delta_{AF}$  Pool-seq vs. GBS

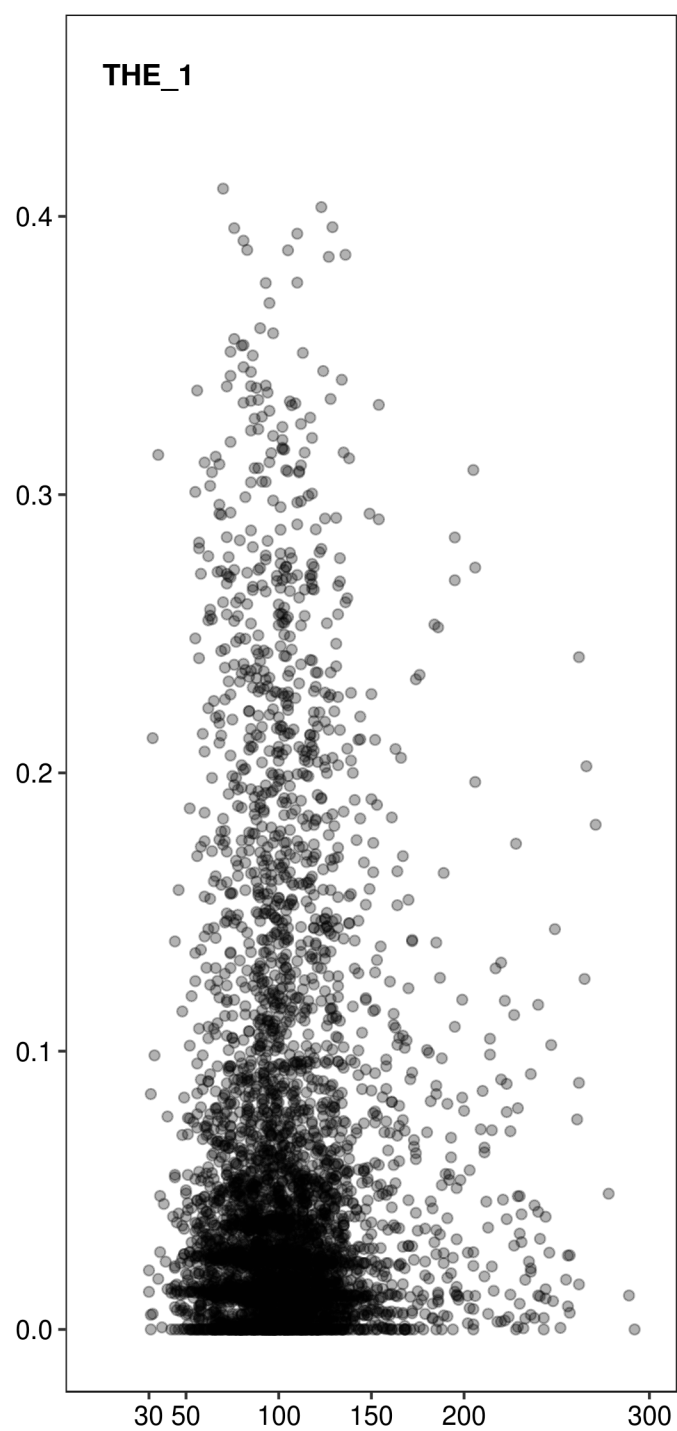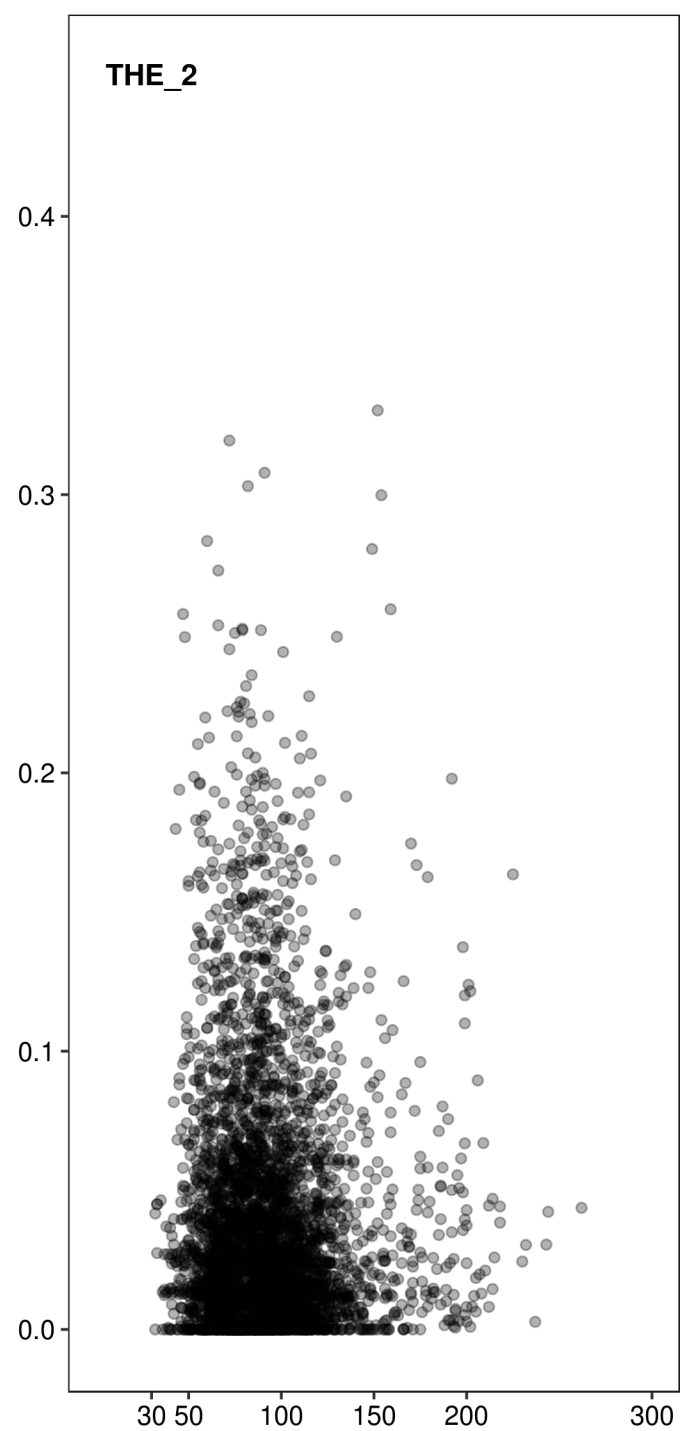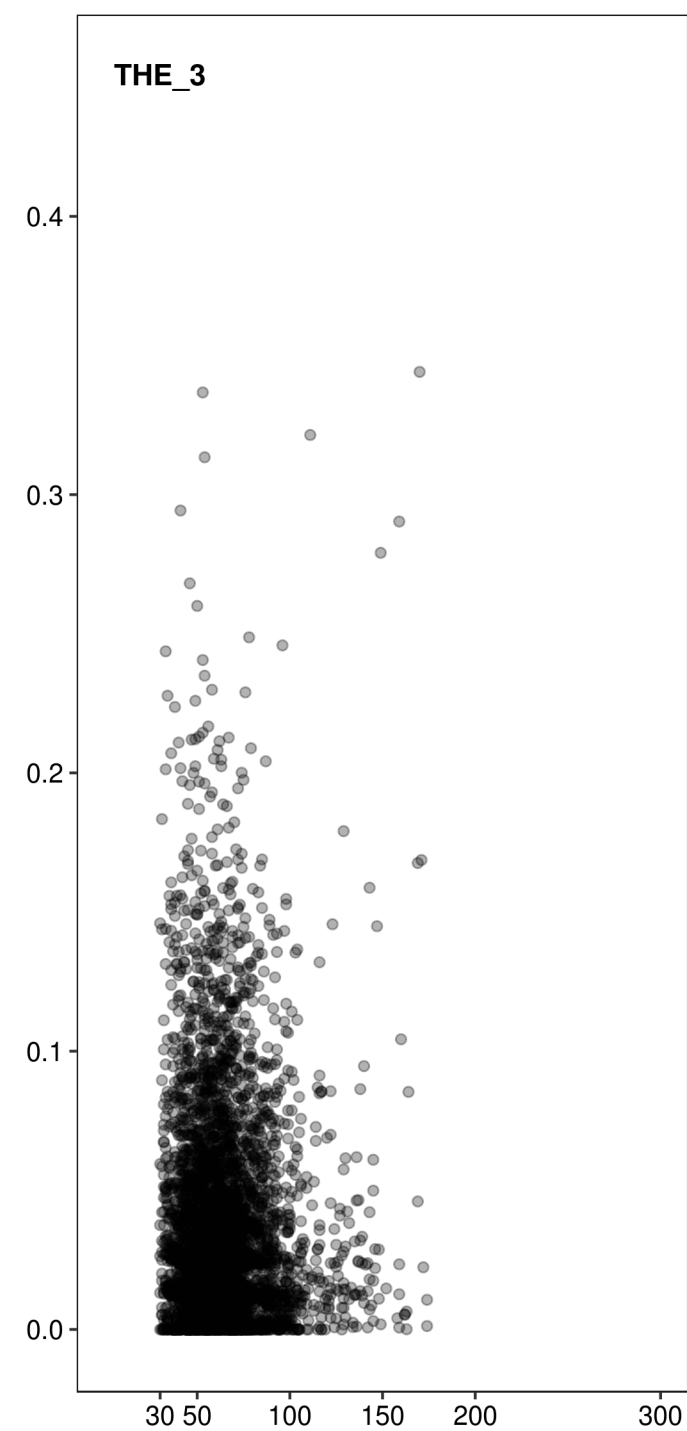

Markers coverage (Pool-seq)

Supplement: Supplementary file 1 [file ECE3-9-6606-s001.zip › ece35240-sup-0001-AppendixS1/ece35240-sup-0007-FigS3.pdf]
